# Supplementary material for: Nucleus-Independent Chemical Shift (NICS) as a Criterion for the Design of New Antifungal Benzofuranones
Source: Molecules. 2021 Aug 21;26(16):5078. doi: 10.3390/molecules26165078 (PMC8401487; doi:10.3390/molecules26165078)
Supplement: Supplementary file 1 [file molecules-26-05078-s001.zip › molecules-1342081-supplementary.pdf]

Article

Nucleus-Independent Chemical Shift (NICS) as a Criterion for the Design of New Antigungal Benzofuranones.

María de los Ángeles Zermeño-Macías, Marco Martín González-Chávez, Francisco Méndez, Arlette Richaud, Rodolfo González-Chávez, Luis Enrique Ojeda-Fuentes, Perla del Carmen Niño-Moreno and Roberto Martínez

Supplementary materials

Reference for MIC in vitro of 6, 7a-g and 8a-g against yeast and filamentous fungus. ….1

Table S1. Equations for simple linear regression NICS (0) and NICS (1) for series I: compounds 6 and 7a-g. ….1

Table S2. Equations for simple regression NICS (0) and NICS (1) for series II: compounds 8a-g. ….2

Table S-3. Summary of Mann-Whitney U test for the comparison of antifungal activity between indol-4-ones and benzofuran-4-ones. ….2

Table S-4. Summary of Mann-Whitney U test for the different comparisons between calculated and experimental MICs. ….3

Atomic coordinates for :

14 ….4
15-1 ….5
15-3 ….6
15-11 ….7
15-15 ….8
15-28 ….9
15-32 …10
15-33 …11
15-34 …12
15-41 …13

The MIC values in vitro of **6**, **7a-g** and **8a-g** against yeast and filamentous fungus were obtained from:

Zermeño-Macías, M.D.l.Á.; González-Chávez, M.M.; Méndez, F.; González-Chávez, R.; Richaud, A. Theoretical Reactivity Study of Indol-4-Ones and Their Correlation with Antifungal Activity. *Molecules* **2017**, *22*, 427. https://doi.org/10.3390/molecules22030427

Table S1. Equations for simple linear regression NICS (0) and NICS (1) for series I: compounds **6** and **7a-g**.

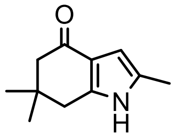

6

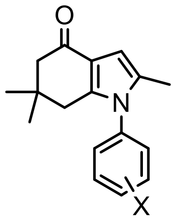

7 a-g

NICS	Microorganism observed	Testing time (h)	Equation	*r*
(0)	*Candica albicans*	24	MIC [mM]= -1.26 mM ppm^{-1}NICS(0) [ppm^{-1}]-12.06 mM	0.70
(0)	*Aspergillus niger*	48	MIC [mM]= -0.75 mM ppm^{-1}NICS(0) [ppm^{-1}]-7.12 mM	0.94
(1)	*Aspergillus niger*	72	MIC [mM]= -1.60 mM ppm^{-1}NICS(0) [ppm^{-1}]-15.22 mM	0.90
	Aspergillus fumigatus	48	MIC [mM]= -0.71 mM ppm^{-1}NICS(0) [ppm^{-1}]-6.75 mM	0.77
	Aspergillus fumigatus	72	MIC [mM]= -1.43 mM ppm^{-1}NICS(0) [ppm^{-1}]-13.50 mM	0.88
	Aspergillus niger	48	MIC [mM]= -0.28 mM ppm^{-1}NICS(1) [ppm^{-1}]-1.52 mM	0.95
(1)	*Aspergillus fumigatus*	72	MIC [mM]= -0.47 mM ppm^{-1}NICS(1) [ppm^{-1}]-2.39 mM	0.91

Table S2. Equations for simple regression NICS (0) and NICS (1) for series II: compounds **8a-g**.

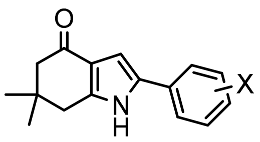

8 a-g

NICS	Microorganism observed	Testing time (h)	Equation	*r*
(0)	*Candica albicans*	48	MIC [mM]= 0.14 mM ppm^{-1} NICS(0) [ppm^{-1}] + 2.32 mM	0.75
	Candida glabrata	24	MIC [mM]= 0.07 mM ppm^{-1} NICS(0) [ppm^{-1}] + 1.16 mM	0.75
	Candida parapsilosis	24	MIC [mM]= -0.05 mM ppm^{-1} NICS(0) [ppm^{-1}] -0.46 mM	0.91
(1)	*Candida albicans*	48	MIC [mM]= 0.29 mM ppm^{-1} NICS(1) [ppm^{-1}] + 2.96 mM	0.83
	Candida glabrata	24	MIC [mM]= 0.14 mM ppm^{-1} NICS(1) [ppm^{-1}] + 1.48 mM	0.83
	Candida tropicalis	48	MIC [mM]= 0.59 mM ppm^{-1} NICS(1) [ppm^{-1}] + 4.45 mM	0.81
	Candida parapsilosis	24	MIC [mM]= -0.09 mM ppm^{-1} NICS(1) [ppm^{-1}] -0.56 mM	0.82

Table S-3. Summary of Mann-Whitney U test for the comparison of antifungal activity between indol-4-ones and benzofuran-4-ones.

Fungi	Testing time	z	Mean ranks		p
			Benzofuran-4-ones	Indol-4-ones	
Yeast	24	4.6533	33.92	15.08	<0.001
	48	1.7235	28.00	21.00	0.0848
Filamentous	48	-2.4735	5.50	11.50	0.0134
	72	-2.4735	5.50	11.50	0.0134

Equality analysis between calculated and experimental MIC data (See Table S-4).

The analyses tested were:
a) Using equations obtained with the simple linear regression from the antifungal activity of indol-4-ones of series I *vs* NICS (0) and NICS (1).
i. With NICS (1): Filamentous fungi 48/72 h, filamentous fungi at 48 h, and filamentous fungi at 72 h.
b) Using equations obtained with the simple linear regression from the antifungal activity of indol-4-ones of series II *vs* NICS (0) and NICS (1).
i. With NICS (0): Yeasts 24 /48 h, yeasts 24 h, yeasts 48 h.
ii. With NICS (1): Yeasts 24/48 h, yeasts 24 h, yeasts 48 h.

A different effect of the calculated and experimental biological activity was found (calculated differently to experimental MIC value) for:
a) Equations obtained by series I
i. NICS (1): Filamentous fungi at 48 and 72 h

b) Equations obtained by series II
i. NICS (1): Yeasts 24 and 48 h.

We found a non-significant effect of the calculated and experimental biological activity (calculated equal to experimental MIC value) for:
a) Equations obtained by series I.
i. NICS (1): Filamentous fungi at 48 h, filamentous fungi at 72 h.
b) Equations obtained by series II
i. NICS (0): Yeasts at 24 and 48 h, yeasts at 24 h, yeasts at 48 h.
ii. NICS (1): Yeasts at 24 h and yeasts at 48 h.

Table S-4. Summary of Mann-Whitney U test for the different comparisons between calculated and experimental MICs.

Series	NICS	Fungi	Testing time	z	Mean ranks		p
					Calculated	Experimental	
I	1	Filamentous	48 and 72 h	2.3630	11.38	5.62	0.0180
			48 h	-1.6290	6.50	10.50	0.1033
			72 h	1.0104	5.50	3.50	0.3123
II	0	Yeast	24 and 48 h	-0.3539	9.00	10.00	0.7234
			24 h	-0.8407	4.60	6.40	0.4005
			48 h	0.000	4.50	4.50	1.000
	1	Yeast	24 and 48 h	-1.9798	13.19	19.81	0.0477
			24 h	-1.2086	7.0	10.0	0.2268
			48 h	-1.6290	6.5	10.50	0.1033

Atomic coordinates for 14

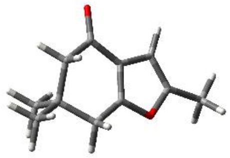

Charge:0 Multiplicity:1

C	-0.49912100	-0.61613900	-0.23765000
C	-0.64935000	0.73529600	-0.10933400
C	0.50909000	1.62586000	-0.14864100
C	1.83062100	0.92471900	-0.47721200
C	0.76045600	-1.37882100	-0.42886800
C	-2.66135100	-0.24006300	0.02103400
C	-2.06005500	0.97407000	0.06332100
H	2.64232900	1.55272400	-0.10220000
H	0.87914900	-1.63169300	-1.49138000
H	-2.54224100	1.92893800	0.19732000
C	1.97555800	-0.52949500	0.04500000
C	3.26711900	-1.14391900	-0.51853900
H	4.14357500	-0.57887500	-0.18842100
H	3.38987700	-2.17694600	-0.17793000
H	3.26215300	-1.14763700	-1.61267100
C	2.05014100	-0.52800200	1.58476300
H	2.19503900	-1.54341700	1.96569700
H	2.89261700	0.08008100	1.92608000
H	1.14275800	-0.12428500	2.04032100
O	0.44490700	2.83062500	0.02497600
O	-1.70255200	-1.23005600	-0.16220100
C	-4.06768100	-0.70513600	0.12795400
H	-4.72722000	0.15101300	0.27520900
H	-4.38352000	-1.23135500	-0.77817800
H	-4.19777900	-1.38947900	0.97200700
H	1.91142700	0.92477400	-1.57315800
H	0.72043100	-2.32861500	0.11414000

Atomic coordinates for 15-1

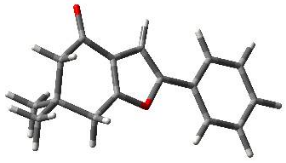

Charge:0 Multiplicity:1

C	0.90139500	-0.45638500	-0.26452200
C	0.95419800	0.90660400	-0.16724900
C	2.23647700	1.61174000	-0.18273800
C	3.44431700	0.71108600	-0.45472000
C	2.03658100	-1.40359900	-0.39714000
C	-1.18754800	0.24594400	-0.08783500
C	-0.40174200	1.35996600	-0.04832300
H	4.33121100	1.21760900	-0.06660300
H	2.14745700	-1.69621800	-1.45025200
H	-0.72536200	2.38296200	0.04959000
C	3.35079700	-0.73458300	0.10141400
C	4.55119300	-1.55113700	-0.40356300
H	5.49221800	-1.11553000	-0.05572700
H	4.50554000	-2.58112500	-0.03572100
H	4.58026700	-1.58470700	-1.49675400
C	3.37531500	-0.70541200	1.64269100
H	3.35306200	-1.72097700	2.04900700
H	4.28824000	-0.22153400	2.00094000
H	2.52501100	-0.15895600	2.05784900
O	2.34808800	2.81532100	-0.03125700
O	-0.37969300	-0.87896800	-0.21915100
C	-2.62525900	0.01713600	-0.02115000
C	-3.15879600	-1.27825000	-0.10925900
C	-3.50797900	1.09924400	0.13338200
C	-4.53416000	-1.48175500	-0.04490300
H	-2.49146300	-2.12206600	-0.22856800
C	-4.87993600	0.88951100	0.19642400
H	-3.11852100	2.10796500	0.20534200
C	-5.40159200	-0.40197200	0.10770800
H	-4.92853500	-2.48933900	-0.11486600
H	-5.54549300	1.73700300	0.31587000
H	-6.47233100	-0.56280800	0.15727500
H	3.55560600	0.67154000	-1.54728800
H	1.83636800	-2.32348500	0.16186100

Atomic coordinates for 15-3

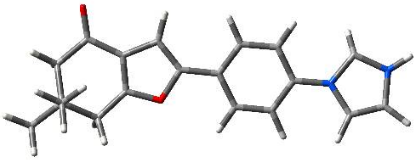

Charge:0 Multiplicity:2

C	-2.61796600	-0.43550200	-0.28217100
C	-2.74728900	0.91908900	-0.15039900
C	-4.06813600	1.54565500	-0.13060700
C	-5.22695500	0.58197400	-0.40479200
C	-3.69657600	-1.44529500	-0.42354700
C	-0.56712400	0.38334200	-0.11534200
C	-1.41816600	1.44954600	-0.03904800
H	-6.13412400	1.02648100	0.01184900
H	-3.43405000	-2.36505200	0.10951300
H	-1.15346500	2.48739700	0.07667100
C	-5.03978100	-0.86930100	0.11155300
C	-5.04037400	-0.88176800	1.65327700
H	-5.97375900	-0.46245900	2.03930800
H	-4.95150700	-1.90492000	2.03073000
H	-4.21609700	-0.29758400	2.06934400
C	-6.19826000	-1.74257500	-0.39652000
H	-6.08506800	-2.77741800	-0.05788100
H	-7.15748000	-1.37339600	-0.02226100
H	-6.24402300	-1.74865100	-1.48966900
O	-4.25144200	2.73735900	0.04914600
O	-1.31269200	-0.78432100	-0.26404500
C	0.87526400	0.23427300	-0.07063400
C	1.49420100	-1.02107100	-0.22286000
C	1.70783000	1.34979800	0.12907000
C	2.87119300	-1.15668800	-0.18370700
H	0.88221800	-1.89928000	-0.38459300
C	3.08523300	1.22807600	0.17228300
H	1.26872200	2.33119200	0.26706900
C	3.69742300	-0.03352700	0.01537700
H	3.30408900	-2.13772900	-0.33244900
H	3.69435000	2.10172900	0.36397900
N	5.08225300	-0.14296700	0.04432300
C	5.82667300	-1.32365700	0.18662700
C	5.97829700	0.94531200	0.01007000
C	7.13785200	-1.00638800	0.14684000
H	5.36305800	-2.28405800	0.31552800
H	5.74294600	1.79245200	-0.62689600
H	7.99613600	-1.64905300	0.24848700
N	7.25705500	0.35645300	-0.06689600
H	8.08129700	0.86962700	0.20337400
H	-5.35704100	0.56337300	-1.49587700
H	-3.80832800	-1.71910800	-1.48185200

Atomic coordinates for 15-11

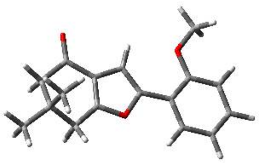

Charge:0 Multiplicity:1

C	-1.38399400	-0.59957500	-0.25737500
C	-1.27362900	0.76150900	-0.19642800
C	-2.46273300	1.61253500	-0.22615200
C	-3.77164600	0.85523800	-0.46932700
C	-2.62384600	-1.40912900	-0.36245400
C	0.77948000	-0.14452700	-0.11095600
C	0.12729700	1.05566300	-0.09704900
H	-4.58873900	1.47331800	-0.08957400
H	-2.53120700	-2.33167800	0.21976100
H	0.58116100	2.02642500	-0.02862800
C	-3.84717300	-0.57782100	0.12192800
C	-3.86122000	-0.50908000	1.66219800
H	-4.70927800	0.08732200	2.01036400
H	-3.95765900	-1.51021800	2.09303200
H	-2.95060800	-0.05717800	2.06286000
C	-5.13841100	-1.25830500	-0.36033800
H	-5.21324800	-2.27761900	0.03180100
H	-6.01944000	-0.70623900	-0.02067900
H	-5.17658100	-1.31348600	-1.45244100
O	-2.43323900	2.82509000	-0.10791200
O	-0.16391900	-1.17048600	-0.20737900
C	2.16824000	-0.57915200	-0.04899900
C	2.48268600	-1.94641600	-0.07804700
C	3.23918900	0.34442200	0.04081000
C	3.79694700	-2.39737300	-0.02093400
H	1.67240800	-2.65999600	-0.14684400
C	4.55752000	-0.11062100	0.09778500
C	4.83522200	-1.47661600	0.06714000
H	4.00461300	-3.46044300	-0.04506600
H	5.37418500	0.59459000	0.16678700
H	5.86528900	-1.81115900	0.11272600
O	2.90268500	1.66428900	0.06671200
C	3.93231000	2.64232100	0.15592200
H	4.50849800	2.53068700	1.08004000
H	4.60526100	2.59226900	-0.70616300
H	3.42395900	3.60503700	0.16152500
H	-3.89566300	0.80224700	-1.55989800
H	-2.77460900	-1.71348300	-1.40732300

Atomic coordinates for 15-15

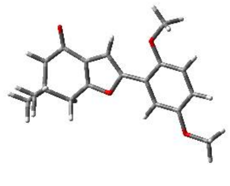

Charge:0 Multiplicity:1

C	-1.77777400	-0.57324500	-0.25093900
C	-1.95175000	0.78231400	-0.20964600
C	-3.29158000	1.36761700	-0.25016600
C	-4.41384100	0.35148000	-0.48308400
C	-2.82298700	-1.62361300	-0.34186800
C	0.24325900	0.32089000	-0.11165000
C	-0.64203500	1.36089700	-0.11461000
H	-5.34335700	0.79243000	-0.11489000
H	-2.54104500	-2.49821900	0.25339700
H	-0.39630100	2.40484000	-0.05938900
C	-4.19269600	-1.05627800	0.13168700
C	-4.22163700	-0.96599500	1.67036100
H	-5.17443900	-0.55111900	2.01115400
H	-4.10857700	-1.95768200	2.11865500
H	-3.42366900	-0.32897700	2.05938200
C	-5.31427800	-1.99683500	-0.33747200
H	-5.17453800	-3.00436700	0.06691600
H	-6.29068000	-1.63603900	-0.00165600
H	-5.34115200	-2.07213400	-1.42872500
O	-3.51529900	2.56115500	-0.14749200
O	-0.46673600	-0.87871500	-0.19305600
C	1.69222500	0.18662200	-0.04523400
C	2.27421100	-1.08323000	-0.07486300
C	2.54626100	1.31585300	0.04902200
C	3.65723600	-1.26026300	-0.01503100
H	1.64714500	-1.96116100	-0.14668700
C	3.92261800	1.13264800	0.10818900
C	4.48754400	-0.14712700	0.07682400
H	4.58356800	1.98529900	0.18012400
H	5.56338500	-0.24507200	0.12457000
O	1.94304500	2.54204400	0.07628800
C	2.75620200	3.70333500	0.17066000
H	3.34249100	3.70618000	1.09590600
H	3.42877500	3.79080800	-0.68948400
H	2.06831300	4.54737200	0.17749300
O	4.09078600	-2.55908700	-0.05364400
C	5.48778200	-2.80224400	-0.00201300
H	5.60461800	-3.88400200	-0.04616100

H	6.00613500	-2.34789800	-0.85419900
H	5.92631300	-2.42938500	0.93073900
H	-4.51859700	0.25672800	-1.57289100
H	-2.90531200	-1.96771900	-1.38202400

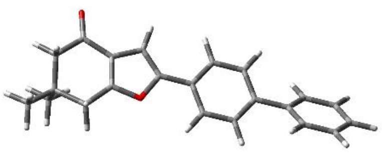

Atomic coordinates for 15-28

Charge:0 Multiplicity:1

C	2.88284400	-0.47913400	-0.20272200
C	3.02564100	0.87903500	-0.27363400
C	4.35242600	1.49196500	-0.34763200
C	5.49990500	0.48793600	-0.48239800
C	3.95319800	-1.50747900	-0.18951700
C	0.84315100	0.37388600	-0.16838800
C	1.70198000	1.43103400	-0.24611000
H	6.41460200	0.97995200	-0.14339400
H	4.05730600	-1.93965400	-1.19417400
H	1.44677000	2.47710800	-0.28383200
C	5.30359100	-0.86777100	0.24772200
C	6.45280300	-1.81689700	-0.12806700
H	7.41601400	-1.40345500	0.18421500
H	6.33334700	-2.78838200	0.36186700
H	6.49399200	-1.98624100	-1.20822700
C	5.31283600	-0.64693100	1.77345900
H	5.22255500	-1.60028900	2.30253300
H	6.25066700	-0.17909400	2.08586300
H	4.49435300	-0.00167500	2.10161000
O	4.54262000	2.69522100	-0.34282800
O	1.57527700	-0.80881500	-0.13832700
C	-0.60593300	0.24941700	-0.11531800
C	-1.22982300	-1.00570600	-0.04767300
C	-1.42143300	1.39274500	-0.13214300
C	-2.61438100	-1.10892800	-0.00050600
H	-0.62357900	-1.90223600	-0.02321700
C	-2.80293200	1.28225600	-0.08309700
C	-3.43324600	0.02904400	-0.01693900
H	-3.06713200	-2.09102700	0.07512500
H	-3.40601100	2.18211800	-0.12220000
H	-0.97161300	2.37682300	-0.19410100
C	-4.91124800	-0.08602500	0.03352900
C	-5.67939800	0.80656800	0.79675200
C	-5.58069800	-1.09178800	-0.68040900
C	-7.06643300	0.69644400	0.84567300
H	-5.18369500	1.57676400	1.37683000
C	-6.96769200	-1.20182300	-0.63217800

H	-5.01120200	-1.77681000	-1.29824200
C	-7.71726700	-0.30832700	0.13141000
H	-7.63902100	1.39139700	1.44992500
H	-7.46437800	-1.98110100	-1.19963500
H	-8.79729600	-0.39383900	0.16907800
H	3.68412100	-2.33295600	0.47752400
H	5.61966900	0.30422500	-1.55918800

Atomic coordinates for 15-32

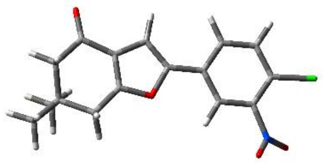

Charge:0 Multiplicity:1

C	-2.24672900	0.31865900	-0.25415000
C	-2.51282100	-1.02007800	-0.16795300
C	-3.89181300	-1.51679800	-0.17285300
C	-4.94496400	-0.43628900	-0.42453900
C	-3.22021100	1.43203200	-0.37094400
C	-0.29685900	-0.70535300	-0.10720500
C	-1.24647400	-1.68368800	-0.06828700
H	-5.06093600	-0.37228400	-1.51547700
H	-3.29156200	1.74228100	-1.42213000
H	-1.08932300	-2.74638200	0.01862100
C	-4.61850000	0.97385800	0.13800600
C	-5.67869600	1.97248300	-0.35347100
H	-6.67318300	1.69195600	0.00446800
H	-5.46539000	2.98117900	0.01364100
H	-5.71345800	2.01167800	-1.44632600
C	-4.63254000	0.93997900	1.67915200
H	-4.43782200	1.93521500	2.08947500
H	-5.60948600	0.61301700	2.04553400
H	-3.88135000	0.25760000	2.08474800
O	-4.18269500	-2.68967700	-0.02690600
O	-0.91381800	0.53333700	-0.21826200
C	1.15619000	-0.70629500	-0.05502700
C	1.87854200	0.49020200	-0.09486300
C	1.87540700	-1.91035400	0.03163500
C	3.26595700	0.47799200	-0.02114500
H	1.37137100	1.44082900	-0.18085600
C	3.26012600	-1.91475300	0.07667800
C	3.98112100	-0.71919400	0.05952100
H	3.80207400	-2.85055600	0.12132500
H	1.34897600	-2.85659100	0.05536300
Cl	5.72004000	-0.79728400	0.06225300

N	3.93210000	1.79919200	-0.02484700
O	3.49580500	2.63429300	-0.80668000
O	4.84296800	1.97842800	0.76682600
H	-2.87360200	2.30617600	0.18905400
H	-5.89617700	-0.80011000	-0.02896100

Atomic coordinates for 15-33

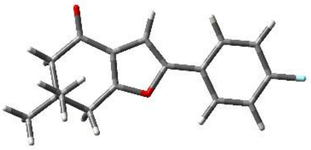

Charge:0 Multiplicity:1

C	-1.28863800	-0.44769200	-0.26788500
C	-1.37047100	0.91340500	-0.17023400
C	-2.66813300	1.59078600	-0.17860800
C	-3.85721500	0.66425700	-0.44487200
C	-2.40341500	-1.41955100	-0.39422800
C	0.78497500	0.30049600	-0.10158200
C	-0.02422900	1.39712700	-0.05817100
H	-4.75318500	1.15129400	-0.05277100
H	-2.18049000	-2.33452300	0.16420700
H	0.27621400	2.42746600	0.03734800
C	-3.72931900	-0.77883800	0.11120200
C	-3.74598100	-0.74970000	1.65256600
H	-4.66707600	-0.28557200	2.01586500
H	-3.69952200	-1.76441000	2.05892300
H	-2.90546700	-0.18488800	2.06314500
C	-4.91434100	-1.62137600	-0.38733300
H	-4.84445800	-2.65009300	-0.01987800
H	-5.86262100	-1.20620800	-0.03431700
H	-4.94869600	-1.65540800	-1.48033600
O	-2.80389600	2.79149700	-0.02561300
O	0.00275700	-0.84194100	-0.22895700
C	2.22720900	0.10145600	-0.04238800
C	2.78778500	-1.18133500	-0.14645200
C	3.08915100	1.19888600	0.12050000
C	4.16569200	-1.36649900	-0.09086000
H	2.13962200	-2.03861400	-0.27225200
C	4.46608700	1.02550000	0.17651900
C	4.98100400	-0.25833700	0.06938800
H	5.13691800	1.86610600	0.30213400
H	2.68240400	2.19922400	0.20603600
H	4.60635900	-2.35241200	-0.17046200
F	6.32381800	-0.43188800	0.12411700

H	-3.97266300	0.62209500	-1.53691300
H	-2.51353800	-1.71502000	-1.44659200

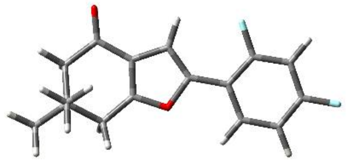

Atomic coordinates for 15-34

Charge:0 Multiplicity:1

C	-1.44954400	-0.52250100	-0.26944200
C	-1.50099500	0.84038600	-0.18014700
C	-2.78527200	1.54538600	-0.19071200
C	-3.99346000	0.64200800	-0.45200400
C	-2.58471300	-1.47134800	-0.38909900
C	0.63976700	0.18166800	-0.11090600
C	-0.14512100	1.29754600	-0.07281300
H	-4.87904200	1.15019100	-0.06330100
H	-2.38045600	-2.38764700	0.17426300
H	0.18585200	2.31717400	0.01763900
C	-3.89620700	-0.79985100	0.11315000
C	-3.91120500	-0.76069400	1.65431000
H	-4.82242000	-0.27548000	2.01488200
H	-3.88548100	-1.77349400	2.06726700
H	-3.05929800	-0.21037100	2.06113500
C	-5.09890300	-1.62052500	-0.37950700
H	-5.05020200	-2.64821800	-0.00575800
H	-6.03796400	-1.18350100	-0.02837200
H	-5.13479800	-1.66053700	-1.47225800
O	-2.89649400	2.74853900	-0.04273700
O	-0.16815400	-0.94598800	-0.22959300
C	2.06998700	-0.07757300	-0.05454000
C	2.58193000	-1.38630700	-0.11922100
C	3.01220300	0.95172200	0.06619600
C	3.94555900	-1.64678500	-0.06614700
H	1.88645000	-2.20920600	-0.21304800
C	4.37744500	0.73264000	0.12204600
C	4.82066000	-0.57834300	0.05387000
H	5.06710800	1.56066900	0.21567600
H	4.32980700	-2.65719800	-0.11660500
F	6.15042000	-0.81434200	0.10676300
F	2.58460600	2.23292600	0.13330200
H	-4.10917100	0.59544200	-1.54382400
H	-2.70121400	-1.77005300	-1.43983100

<u>Atomic coordinates for 15-41</u>

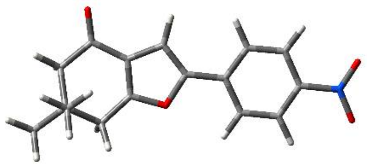

Charge:0 Multiplicity:1

C	-1.94658600	-0.44590000	-0.27116500
C	-2.05744700	0.91482000	-0.17435500
C	-3.37075500	1.56695200	-0.17280600
C	-4.54072200	0.61617600	-0.43161500
C	-3.04340500	-1.43831100	-0.38907500
C	0.10906600	0.34583500	-0.12070900
C	-0.72453700	1.42647900	-0.07261200
H	-4.66078900	0.57007900	-1.52302300
H	-2.79808900	-2.34911600	0.16633900
H	-0.44564600	2.46322700	0.02100000
C	-4.37818100	-0.82329600	0.12613600
C	-4.38256100	-0.79198300	1.66748400
H	-5.31053100	-0.34782400	2.03774400
H	-4.31043000	-1.80461700	2.07497900
H	-3.55177700	-0.20807500	2.07149300
C	-5.54892100	-1.69108100	-0.36243400
H	-5.45448000	-2.71766300	0.00505300
H	-6.50258000	-1.29564400	-0.00176500
H	-5.59162300	-1.72641900	-1.45505400
O	-3.52500800	2.76378300	-0.01605400
O	-0.64890000	-0.81309800	-0.24096100
C	1.55114600	0.18335600	-0.07034500
C	2.13923800	-1.09131600	-0.15624600
C	2.38741500	1.30730000	0.06648200
C	3.51732100	-1.24126500	-0.10753200
H	1.50907500	-1.96415800	-0.26174800
C	3.76402800	1.16649000	0.11570500
H	1.95645000	2.29826900	0.13582900
C	4.31626100	-0.11008500	0.02775200
H	3.97949400	-2.21655300	-0.17270100
H	4.41458500	2.02352800	0.22114800
N	5.77984400	-0.26477500	0.08007400
O	6.45658800	0.75072400	0.19953900
O	6.23703200	-1.39978600	0.00110500
H	-5.44480600	1.08437400	-0.03560100
H	-3.15360100	-1.73399200	-1.44122100
